# Peri- and Intraarticular Injections with Isolable Treatment Effects in Recurrent Mandibular Dislocation: A Mapping Review of the Current Evidence

**DOI:** 10.3390/jcm15145589

**Published:** 2026-07-16

**Authors:** Amelia Hoppe, Maciej Chęciński, Wojciech Macek, Maja Kosińska, Karolina Grzybowska-Kowalczyk, Tomasz Horodniczy, Julia Kasprzycka, Oliwia Jagiełło, Zuzanna Baniak, Kamila Chęcińska, Maciej Sikora

**Affiliations:** 1 Department of Oral Surgery, Preventive Medicine Center, Komorowskiego 12, 30-106 Cracow, Poland; amelia.a.hoppe@gmail.com (A.H.); ld.wojciech.macek@wp.pl (W.M.); majaakosinska@gmail.com (M.K.); 2 National Medical Institute of the Ministry of the Interior and Administration, Wołoska 137, 02-507 Warsaw, Poland; maciej.checinski@pimmswia.gov.pl (M.C.); maciej.sikora@pimmswia.gov.pl (M.S.); 3 Department of Maxillofacial Surgery, Hospital of the Ministry of the Interior and Administration, Wojska Polskiego 51, 25-375 Kielce, Poland; 4 Uśmiech Family Dental Clinic, Nastrojowa 26, 91-496 Łódź, Poland; karolina.orto.gk@gmail.com; 5 Ortho.pl Dental Office, Buforowa 34, 52-131 Wrocław, Poland; tomasz.horodniczy2@gmail.com; 6 Miodowa Clinic, Głowaccy Medical and Dental Practice Professional Partnership, Miodowa 2, 62-090 Kiekrz, Poland; 7 Gabinety Lekarskie Dent-im M.B. Pawelczyk Spółka Jawna, Różana 13/1, 61-577 Poznań, Poland; oliwiajagiellodent@gmail.com; 8 Stomatologia Centrum, Moniuszki 3/7, 32-020 Wieliczka, Poland; baniakzuzanna@gmail.com; 9 Department of Biochemistry and Medical Chemistry, Pomeranian Medical University, Powstańców Wielkopolskich 72, 70-111 Szczecin, Poland

**Keywords:** recurrent mandibular dislocation, temporomandibular joint, TMJ dislocation, autologous blood injection, dextrose prolotherapy, sclerosing agents, sodium morrhuate, intra-articular injection, periarticular injection, mapping review

## Abstract

**Background/Objectives:** Recurrent temporomandibular joint dislocation is associated with repeated dislocation episodes, pain, impaired jaw function, and psychosocial burden. Injectable intra- and periarticular therapies have been proposed as minimally invasive methods of improving joint stability, but the available evidence is heterogeneous and frequently involves adjunctive procedures. This mapping review aimed to characterize injectable treatments whose effects could be assessed independently from simultaneous non-injectable interventions. **Methods:** PubMed (MEDLINE), Europe PMC, and BASE were searched from inception to 7 April 2026. Reference lists of included studies and relevant reviews were also screened. Studies reporting clinical outcomes of injectable intra- or periarticular treatments for recurrent mandibular dislocation were eligible when the effect of the injectable component could be evaluated independently. Study selection, data charting, and critical appraisal were performed using predefined methods. **Results:** Five primary clinical studies and eight secondary mapping or reference-checking sources were included. The primary studies evaluated autologous blood injection, dextrose prolotherapy, and sodium morrhuate or other sclerosing-agent injections. Most studies reported reductions in recurrent dislocation or subluxation and improvements in joint stability. Some also reported improvements in maximal mouth opening, clicking, pain, or other clinical outcomes. However, the evidence was limited by small sample sizes, heterogeneous protocols, variable injection sites, and limited comparative data. **Conclusions:** The available literature on injectable therapies for recurrent mandibular dislocation is limited and heterogeneous. Although included studies generally reported favorable outcomes, the evidence does not permit conclusions regarding the comparative effectiveness or superiority of any specific injectable modality.

## 1. Introduction

### 1.1. Rationale

**Figure 1:**
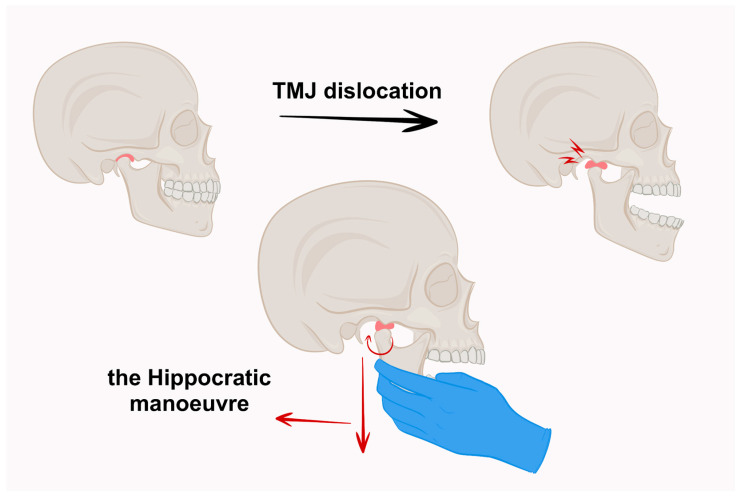
Schematic illustration of TMJ dislocation and manual reduction, with arrows indicating the directions of mandibular movement. Original illustration created by the co-author (A.H). Created in Procreate v. 5.4 (Savage Interactive, 2026), for iPadOS.

Mandibular dislocation represents a clinically challenging condition associated with pain, impaired jaw function, and considerable psychosocial burden for affected patients [1,2,3]. It typically occurs when, during translational movement over the articular eminence, excessive excursion prevents the mandibular condyle from returning to the glenoid fossa. The Hippocratic maneuver involves placing the thumbs on the occlusal surfaces of the mandibular molars and securing the mandible with the remaining fingers to execute controlled repositioning motion (Figure 1). Although manual reduction provides immediate symptom relief following an acute dislocation, patients with recurrent episodes should be considered for interventions that provide long-term stability [4,5]. Conservative management approaches, such as physical therapy and behavioral modifications, rely heavily on patients’ cooperation and commitment, yet may still prove insufficient when addressing persistent, habitual dislocation [6,7]. In such cases, minimally invasive injectable therapies have gained increasing attention as alternatives to surgical intervention [4,6].

Injectable management of recurrent mandibular dislocation includes both intra-articular and peri-articular approaches [8,9]. A variety of agents have been described, including hypertonic dextrose and autologous blood products [4,10,11]. These therapies may be administered either into periarticular tissues to promote capsular fibrosis or reduce muscular hyperactivity, thereby enhancing joint stability, or directly into the intra-articular superior joint space of the temporomandibular joint (TMJ), targeting different pathophysiological mechanisms [8,12,13].

The current literature on injectable therapies for recurrent mandibular dislocation is heterogeneous and methodologically diverse. Published studies vary considerably with regard to injected substances, anatomical targets, procedural techniques, dosing regimens, treatment schedules, patient selection criteria, and outcome measures [4,12,14]. This diversity makes direct comparison between studies difficult and limits the ability to determine which therapeutic strategies are most effective in specific clinical contexts [12,14].

Given this complexity, a mapping review of the available evidence is warranted. Such an approach enables identification and categorization of injectable interventions according to both administered substance and target site, including intra-articular and periarticular techniques. It can also clarify patterns in study design, reported outcomes, and current research priorities, while highlighting important evidence gaps. By providing a structured overview of the field, this review may support evidence-informed clinical decision-making and help guide future comparative and higher-level research.

### 1.2. Objectives

The objective of this review was to provide a systematic map of the current evidence on injectable intra- or periarticular substances used in the management of recurrent mandibular dislocation. The review aimed to catalogue the range of agents investigated, the clinical settings in which they were applied, the study designs used, and the outcomes reported. In addition, this review sought to identify patterns in the existing evidence base and to indicate underexplored areas that may warrant further comparative or higher-level clinical research.

To improve interpretability, this review specifically focused on injectable interventions for which the clinical effect of the injectable component could be considered independently from adjunctive immobilization, surgery, arthroscopy, or other simultaneous non-injectable procedures.

## 2. Materials and Methods

### 2.1. Protocol and Registration

The review protocol was prospectively registered in the Open Science Framework (OSF) under registration number osf.io/p3tm5. The review was designed and conducted as a mapping review. A mapping review was chosen because the available evidence was sparse and heterogeneous, precluding meaningful quantitative comparison, and the aim was to characterize the evidence base rather than assess comparative effectiveness. A PRISMA 2020 flow diagram was used only to visualize the study selection process [15].

### 2.2. Eligibility Criteria

The predefined inclusion and exclusion criteria are described below and summarized in Table 1.

This mapping review included studies of any design that reported on injectable substances used to prevent or manage recurrent mandibular dislocation (recurrent temporomandibular joint/mandibular dislocation). Eligible participants were patients of any age with a diagnosis of recurrent mandibular dislocation as defined by the study authors (clinical history of recurrent episodes, with or without imaging confirmation). Studies exclusively addressing single acute dislocations without data on recurrence, or studies of other TMJ disorders without stratified data for recurrent dislocation, were excluded.

Interventions comprised any injectable agent administered intra-articularly or peri-articularly with the intent to stabilize the joint or reduce recurrence, including but not limited to autologous blood products (e.g., whole autologous blood, platelet-rich plasma), hyaluronic acid and other fillers, hypertonic solutions (e.g., dextrose), corticosteroids, botulinum toxin, bone substitutes (e.g., hydroxyapatite), fibrin or coagulation-promoting agents, and other biologics or experimental injectables. Studies describing injection techniques, dosing regimens, or procedural details in the context of clinical outcomes were included. Studies in which injections were combined with other simultaneous interventions (surgical or non-injectable) were excluded unless the effect of the injectable component could be clearly isolated. This decision was made intentionally to improve interpretability of intervention-specific evidence mapping.

Comparators of any type were eligible, including placebo/sham, no injection, conservative management, alternative injectables, or surgical treatments; single-arm studies, case series, and case reports were also included to comprehensively map the evidence landscape. Eligible outcomes included measures of recurrence (frequency of dislocation episodes, time to recurrence), functional outcomes (maximum interincisal opening, jaw function), pain scores, patient-reported outcomes and satisfaction, need for subsequent interventions (including surgery), and adverse events or complications related to the injection.

All study designs reporting primary clinical data were eligible: randomized controlled trials, controlled clinical trials, cohort studies (prospective and retrospective), case–control studies, case series, and case reports. Systematic reviews and narrative reviews were included for mapping purposes but not counted as primary evidence; preclinical animal or in vitro studies were excluded. Conference abstracts were considered only if sufficient data were available in the abstract or via contact with authors.

No language restrictions were applied; non-English full texts were translated where feasible. The search covered all publication years up to the date of search closure, 7 April 2026.

### 2.3. Information Sources

We searched three electronic bibliographic databases from inception to the date of search closure: PubMed (MEDLINE), Europe PMC, and BASE. Reference lists of included studies and relevant reviews were screened for additional records. Where necessary, corresponding authors were contacted to obtain full texts or clarifying data. The date of the final search is reported in the Methods.

Searches for primary research reports were conducted primarily in medical databases. The query was formulated based on the eligibility criteria specified above. It was refined during preliminary searches. The following query was finally used in all search engines:

(“temporomandibular joint” OR “TMJ” OR temporomandibular OR mandibular OR mandible) AND (dislocation OR dislocations OR dislocated OR recurrent OR recurrently OR recurrent*) AND (inject* OR “intra-articular” OR intraarticular OR “peri-articular” OR periarticular OR “injection therapy” OR injection) AND (dextrose OR “hypertonic dextrose” OR “autologous blood” OR “platelet rich plasma” OR PRP OR “hyaluronic acid” OR hyaluronate OR HA OR steroid* OR corticosteroid* OR “botulinum toxin” OR botulinum OR hydroxyapatite OR filler* OR fibrin OR biologic* OR “blood product*” OR “autologous blood”).

### 2.4. Selection of Sources

All records identified through the database search were imported into Rayyan, where duplicates were removed (T.H.). The selection of sources of evidence was conducted in two stages using Rayyan. Before formal screening, the reviewers performed a calibration exercise to ensure consistent application of the eligibility criteria. In the first stage, titles and abstracts were independently and blindly screened by two reviewers (J.K. and O.J.) to identify potentially relevant reports. In the second stage, the full texts of potentially eligible articles were independently assessed by the same reviewers against the predefined inclusion and exclusion criteria. Any disagreements were resolved through discussion and consensus. Reasons for exclusion at the full-text stage were recorded. The selection process was presented in a flow diagram.

### 2.5. Data Charting Process

Data from the included sources of evidence were charted using a standardized data charting form developed by the review team. The form was pilot-tested on a small sample of eligible articles and refined iteratively as needed. Two reviewers (J.K. and O.J.) independently charted the data using structured tables prepared in Google Sheets to enhance consistency and accuracy. Any discrepancies were resolved through discussion and consensus between the reviewers.

Terminology was harmonized during data charting. Studies using terms such as habitual dislocation or habitual luxation were classified under recurrent mandibular/TMJ dislocation when the clinical description indicated repeated dislocation episodes.

### 2.6. Data Items

The following data items were charted from each included source of evidence using a predefined data charting form: bibliographic details, study characteristics, study design, clinical setting, characteristics of the study population, the injectable substance investigated, details of its administration, reported outcomes, and main findings. Additional relevant information was recorded when available.

### 2.7. Critical Appraisal of Individual Sources of Evidence

A critical appraisal was conducted to assess the methodological limitations of the included primary studies and to support a cautious interpretation of the mapped evidence. Included studies were methodologically heterogeneous, including prospective case series and one prospective double-blind comparative study. The appraisal was conducted using the Joanna Briggs Institute’s critical appraisal tools, including the JBI checklists for case series and quasi-experimental studies, as appropriate. The focus was on assessing the clarity of the inclusion criteria, the accuracy of the diagnosis and outcome measures, the reporting of participant characteristics, the adequacy of the statistical analysis, and the transparency of the intervention reporting. The methodological quality of the included studies was varied and generally low to moderate.

### 2.8. Synthesis of Results

The synthesis of results was performed using a structured descriptive mapping approach. Extracted data were grouped according to the injectable substance investigated, anatomical deposition site (intra-articular, periarticular, or combined approaches), study design, and reported clinical outcomes.

The mapped evidence was subsequently organized into summary tables to enable comparison of the investigated interventions, procedural characteristics, and direction of reported findings across the included studies. Particular attention was given to recurrence-related outcomes, mandibular function, pain, follow-up duration, and intervention protocols.

No quantitative pooling or meta-analytic synthesis was performed because of the substantial heterogeneity of the available evidence regarding study design, patient populations, injectable agents, comparator use, procedural techniques, and outcome reporting. Instead, the synthesis focused on identifying recurring patterns within the literature, differences between injectable modalities, and areas where evidence remains limited or insufficiently explored.

## 3. Results

### 3.1. Selection of Sources of Evidence

Five primary clinical studies and eight secondary mapping or reference-checking sources were included. Inter-rater agreement between the two reviewers was assessed using Cohen’s kappa coefficient. Substantial agreement was observed at both the title/abstract screening stage (κ = 0.89) and the full-text review stage (κ = 0.92).

At the title and abstract screening stage, 231 records were excluded because they clearly did not meet the predefined eligibility criteria, including ineligible populations, interventions, clinical conditions, study designs, or absence of relevant clinical outcome data. Individual exclusion reasons were not recorded at this preliminary screening stage.

**Figure 2:**
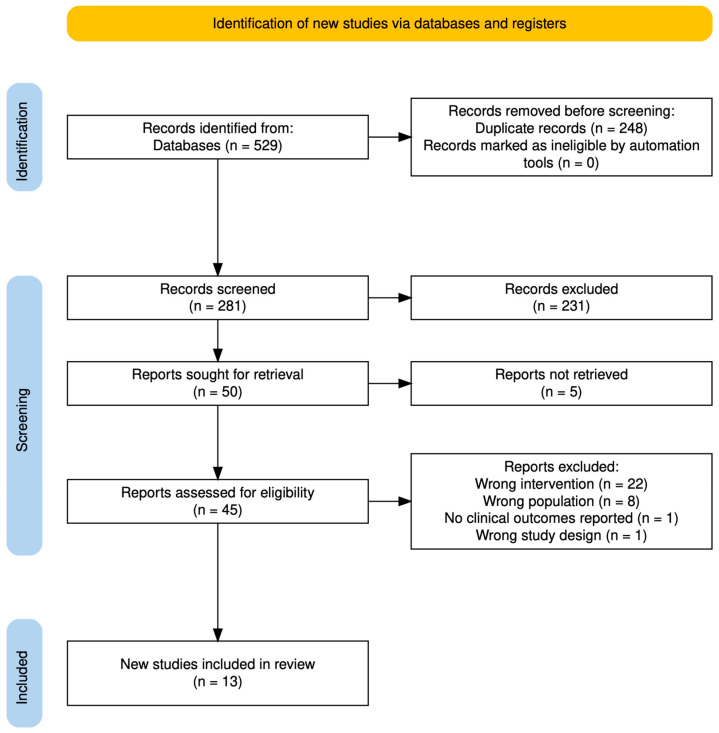
Flow diagram of the study selection process [16].

Reports excluded after full-text assessment or supplementary eligibility clarification are presented in Table A1. The overall study selection process is illustrated in a flow diagram (Figure 2). The final set of included sources represents the body of evidence available on injectable substances used in the management of recurrent mandibular dislocation.

### 3.2. Characteristics of Sources of Evidence

The included sources of evidence consisted of primary clinical studies and secondary sources used for mapping and reference checking (Table A2). Primary clinical studies provided patient-level data on injectable substances used for recurrent mandibular dislocation and were used as the main basis for intervention-level mapping. Secondary sources, including review articles, systematic reviews, meta-analyses, overviews, and dissertations, were considered separately and were used to contextualize the evidence base and support reference checking.

Primary clinical studies were extracted as the main source of patient-level evidence. The data charting process was designed to map the range of injectable substances used for recurrent mandibular dislocation and to summarize how these interventions were reported across the available clinical literature. Extracted items included author and year of publication, study design, population, sample size, injectable substance, injection site, follow-up period, outcomes, and the authors’ main findings. When relevant methodological or procedural details were not clearly reported in the original study, they were recorded as not available or not clearly reported.

Across the primary clinical studies, the investigated interventions were primarily autologous blood injection (ABI), dextrose prolotherapy, and sclerosing agent injection. The studies varied in design, sample size, injection site, follow-up duration, and reported outcomes, which most commonly included recurrence of dislocation, maximal mouth opening, pain, functional improvement, and treatment success (Table A2).

Secondary sources were charted separately from primary clinical studies. These sources included narrative reviews, systematic reviews, meta-analyses, overviews, and dissertations relevant to injectable or minimally invasive management of recurrent mandibular dislocation and related conditions. They were used to provide context for the available evidence, identify previously discussed injectable interventions, and support reference checking (Table A3).

### 3.3. Critical Appraisal Within Sources of Evidence

Most studies clearly described the injectable agent, injection site, duration of follow-up, and primary clinical outcomes, particularly dislocation recurrence, maximum mouth opening, pain, and adverse events [17,18,19,20,21]. However, significant limitations included small sample sizes, lack of randomization in most studies, lack of control groups, incomplete reporting of blinding procedures, and heterogeneity in outcome assessment. Detailed bias risk assessment is presented in Table A4 and Table A5.

### 3.4. Results of Individual Sources of Evidence

The results of individual primary sources of evidence are summarized in Table 2. The table maps the intervention comparisons and direction of findings reported across the included primary studies. Because several studies did not include an active or placebo comparator, some entries represent single-arm intervention evidence rather than comparative effectiveness data [17,19,20,21]. For each source, the table presents the injectable intervention or comparison, anatomical deposition site, volume per administration, number of administrations, total administered volume when calculable, and the authors’ overall conclusion.

Overall, the included primary studies most commonly evaluated ABI, followed by dextrose prolotherapy and sodium morrhuate injection. The direction of findings was generally favorable, with studies reporting reduced recurrence of dislocation or subluxation episodes after injectable treatment. However, interpretation was limited by small sample sizes, heterogeneous injection protocols, variable deposition sites, and the frequent absence of comparator groups.

### 3.5. Synthesis of Results

The available evidence on injectable therapies for recurrent mandibular dislocation was characterized by substantial heterogeneity with respect to study design, injectable agents, administration techniques, follow-up duration, and reported outcome measures. Most included primary studies investigated ABI, whereas fewer studies evaluated dextrose prolotherapy or sclerosing agents such as sodium morrhuate.

Across the included studies, the reported outcomes were generally favorable, particularly regarding reduction of recurrent dislocation episodes and improvement in joint stability. Several studies additionally reported improvement in maximal mouth opening, clicking, and patient-reported functional outcomes following treatment. However, direct comparison between interventions remained difficult because of differences in injection protocols, anatomical deposition sites, comparator use, and treatment schedules.

Both intra-articular and periarticular approaches were represented among studies reporting reductions in recurrent dislocation episodes and other favorable clinical outcomes. One comparative study reported numerically more favorable outcomes following combined intra- and periarticular administration than following periarticular injection alone, although the difference was not statistically significant [18].

Overall, the current body of evidence remains limited by the predominance of case series and small clinical studies, heterogeneous methodologies, and the frequent absence of comparator groups. Although most included studies reported favorable post-treatment outcomes, the available evidence remained insufficient to determine the superiority of any specific injectable modality for recurrent mandibular dislocation.

## 4. Discussion

### 4.1. Summary of Evidence

The currently available evidence regarding injectable therapies for recurrent mandibular dislocation remains limited and methodologically heterogeneous. Most published clinical studies focused on blood-derived therapies, dextrose prolotherapy and sclerosing agent injection, whereas evidence regarding other injectable approaches remains relatively sparse and is supported mainly by isolated reports and secondary evidence sources [11,13,22]. Across the included primary studies, the most frequently investigated interventions were ABI, dextrose prolotherapy, and sclerosing agent injection [17,18,19,20,21]. Reported outcomes were generally favorable, particularly in relation to reduction of recurrent dislocation episodes and improvement in joint stability [17,18,19,20,21]. However, the evidence base was composed mainly of case series, with limited comparative data, which substantially restricts the strength and generalizability of the conclusions [11,22].

Among the mapped injectable approaches, blood-derived therapies were the most frequently investigated interventions. Studies of both intra-articular and periarticular administration reported reductions in recurrence and improvements in selected functional outcomes [18,20,21]. One study compared combined intra- and periarticular blood administration with periarticular injection alone, but did not demonstrate statistically significant superiority [18]. Previous systematic reviews similarly concluded that ABI may represent a promising minimally invasive option for recurrent TMJ dislocation and hypermobility, while simultaneously emphasizing the low overall quality and heterogeneity of the currently available evidence [11,22].

Dextrose prolotherapy represented another injectable modality identified in the mapped evidence. In the included primary evidence, dextrose injection was associated with reduced dislocation or subluxation episodes and improvement in clinical symptoms [17]. Secondary evidence, including systematic reviews and meta-analyses, has also suggested potential benefits of dextrose prolotherapy in TMJ hypermobility, subluxation, or dislocation-related conditions [23,24]. Nevertheless, many prolotherapy studies included mixed populations with TMJ hypermobility, subluxation, or broader temporomandibular disorders rather than clearly defined recurrent mandibular dislocation populations [24,25]. This limits the direct applicability of these findings to recurrent mandibular dislocation specifically.

Clinical evidence regarding sclerosing agent injection remained limited. Although the available data suggested favorable clinical outcomes after sodium morrhuate injection, this evidence was derived from older, non-comparative clinical data and should therefore be interpreted with caution [19]. This finding further illustrates the uneven distribution of evidence across injectable modalities.

The investigated agents are based on distinct biological rationales. The proposed mechanism of autologous blood injection involves a localized inflammatory response, clot formation, and subsequent periarticular fibrosis, which may limit excessive condylar translation [11,20]. Hypertonic dextrose prolotherapy is proposed to stimulate a controlled inflammatory and reparative response in periarticular tissues, whereas sodium morrhuate is used as a sclerosant to induce periarticular fibrosis and thereby enhance joint stability [19,23,26]. These mechanisms remain largely theoretical in the context of recurrent mandibular dislocation, and the magnitude of the clinical response may depend on the injection site, administered volume and concentration, number of sessions, baseline joint characteristics and severity of instability, and use of adjunctive immobilization [4,12,14]. Such factors may contribute to the variability in reported outcomes and limit direct comparison between treatment modalities.

The mapped evidence demonstrated marked heterogeneity in procedural protocols and outcome reporting, including differences in injected substances, administration techniques, injection sites, treatment schedules, follow-up duration, and definitions of therapeutic success. Such methodological diversity limits direct comparison between studies and currently prevents formulation of standardized therapeutic recommendations [22]. In addition, several clinically important prospective and randomized studies were excluded because injections were combined with temporary mandibular immobilization or other adjunctive procedures, preventing the independent contribution of the injectable component from being determined [11,22]. Because immobilization is often incorporated into the therapeutic protocol itself, this exclusion strategy may have reduced the clinical representativeness of the review. The present review should therefore be interpreted as a focused map of intervention-specific evidence rather than a comprehensive review of all injection-based treatment protocols.

Previous reviews have also highlighted the lack of high-quality comparative trials evaluating injectable therapies against surgical approaches or standardized conservative management [4,14,22]. Consequently, despite generally favorable reported outcomes, the current body of evidence should still be interpreted cautiously. Consequently, despite generally favorable reported outcomes, the current evidence should be interpreted cautiously and does not support broad clinical recommendations. Further prospective controlled studies using standardized protocols and clearly defined recurrent mandibular dislocation populations remain necessary [4,14,22].

### 4.2. Strengths

This review has several important strengths. It provides an organized and up-to-date summary of the literature addressing the use of injectable substances for recurrent mandibular dislocation, encompassing therapeutic agents, anatomical targets, study designs, and reported outcomes.

A systematic and transparent approach guided the selection and analysis of studies, including predefined eligibility criteria, structured searches of multiple databases, independent screening, structured data extraction, and critical appraisal using appropriate JBI tools. Independent verification by several reviewers enhanced methodological reliability and reduced the risk of bias.

By mapping the available evidence, the review identifies consistent patterns, differences across treatment approaches, and important gaps requiring further investigation. The distinction between primary clinical evidence and secondary mapping or reference-checking sources additionally improved the clarity of evidence interpretation. This structured synthesis offers valuable insights for clinicians and may help guide the design of future comparative and higher-quality studies exploring injectable modalities for recurrent mandibular dislocation.

### 4.3. Limitations

This review has several limitations. The included studies were heterogeneous in terms of design, patient populations, injectable agents, procedural techniques, injection sites, follow-up, and outcome measures, which limited direct comparison and precluded quantitative synthesis. Most primary studies were small and non-comparative, reducing the reliability and generalizability of the findings.

Interpretation was further complicated by inconsistent terminology, particularly the overlapping use of recurrent dislocation, habitual dislocation, subluxation, and TMJ hypermobility. Some studies were excluded because they involved mixed populations, combined interventions, or outcomes that did not allow the effect of the injectable component to be assessed independently. This included several clinically relevant prospective and randomized studies using adjunctive mandibular immobilization. Because immobilization may form part of the treatment protocol itself, this exclusion strategy narrowed the clinical scope of the review and may have introduced selection bias, although it improved the interpretability of intervention-specific findings.

## 5. Conclusions

The available evidence on injectable therapies for recurrent TMJ dislocation remains preliminary and highly heterogeneous. The included studies evaluated autologous blood, dextrose, and sclerosing agents and generally reported reductions in recurrence. These findings were derived mainly from small, uncontrolled studies with variable protocols and outcome definitions. The current evidence does not permit conclusions regarding comparative effectiveness or support broad clinical recommendations.

## Figures and Tables

**Table 1:** Eligibility criteria.

Category	Inclusion	Exclusion
Population	Patients of any age with recurrent mandibular dislocation as defined by study authors	Studies of only single/acute dislocation or other TMJ disorders without stratified data for recurrent dislocation
Interventions	Any intra-articular or peri-articular injectable intended to stabilize joint or reduce recurrence (e.g., autologous blood/PRP, hyaluronic acid/fillers, hypertonic dextrose, corticosteroids, botulinum toxin, hydroxyapatite, fibrin/coagulation agents, other biologics/experimental injectables). Includes studies describing injection technique/dosing with clinical outcomes	Studies where injections are combined with simultaneous interventions (surgical/non-injectable) without ability to isolate injectable effect
Comparators	Any comparator including placebo/sham, no injection, conservative care, alternative injectables, surgical treatments; single-arm studies, case series, and case reports included for mapping	
Outcomes	Recurrence metrics (frequency, time to recurrence), functional outcomes (MIO, jaw function), pain scores, patient-reported outcomes/satisfaction, need for subsequent interventions (including surgery), adverse events/complications, procedural details	Studies without any clinical outcome data for recurrent dislocation
Study designs	RCTs, controlled clinical trials, cohort studies (prospective/retrospective), case–control, case series, case reports; systematic and narrative reviews included for mapping (not as primary evidence)	Animal studies; in vitro studies
Timeframe	No date restrictions (date of last search: 7 April 2026)	–
Language	No language restrictions; non-English reports included if data extractable or obtainable via translation/author contact	Full texts unavailable and not translatable/assessable

**Table 2:** Intervention comparisons and direction of findings across primary studies.

First Author, Year	Comparison	Injection/Deposition Site	Volume per Administration	Number of Administrations	Total Volume Administered	Authors’ Overall Conclusion
Zhou et al., 2014 [17]	50% dextrose, no comparator	Posterior periarticular tissues around the condylar neck; the joint space was not entered	2 mL of 50% dextrose, preceded by 2 mL of 2% lignocaine	1–3 sessions, depending on clinical response	Depending on the number of sessions: 2–6 mL of 50% dextrose; 4–12 mL total injected fluid, including lignocaine	The authors reported reduced dislocation/subluxation episodes and improvement in clicking. Most successful cases required only one injection.
Machon et al., 2018 [18]	Intra-articular + periarticular autologous blood vs. periarticular autologous blood only	Group A: superior articular cavity plus periarticular/extra-articular tissues during needle withdrawal. Group B: periarticular/extra-articular injection into the capsule only; no blood was injected into the joint cavity.	Group A: 2 mL into the superior joint cavity + 1 mL periarticularly. Group B: 1 mL periarticularly	One injection session	Group A: 3 mL; Group B: 1 mL	Intra-articular plus periarticular ABI was considered more effective than periarticular blood application alone, although the difference was not statistically significant.
Oshiro et al., 2014 [20]	Autologous blood injection, no therapeutic comparator	Superior joint compartment plus pericapsular tissues, including anterior, posterior, and external pericapsular areas	Approximately 3 mL into the superior compartment + approximately 2 mL into pericapsular tissue	One injection session	Approximately 5 mL	No recurrent TMJ dislocations were reported during 1-year follow-up. The authors concluded that ABI around the TMJ capsule was effective and safe.
Yoshida et al., 2018 [21]	Autologous blood, no comparator	According to the Oshiro et al. protocol; likely superior joint compartment plus pericapsular tissues	Not explicitly reported in this article; treatment was performed as described by Oshiro et al.	1–3 injection sessions within 36 months, depending on recurrence	Not explicitly reported; dependent on the number of sessions. Oshiro et al. used approximately 5 mL per session.	The authors concluded that ABI was safe and effective for recurrent TMJ dislocation, especially in patients unsuitable for mouthpiece therapy or surgery.
Xu et al., 1992 [19]	5% sodium morrhuate/sclerosing agent, no comparator	Extra-articular injection around the TMJ capsule/condylar neck region; not injected into the joint cavity	0.5 mL per injection point; approximately 0.5–1 mL per treatment session	1–3 sessions depending on clinical response	0.5–3 mL depending on the number of sessions	The authors reported high clinical effectiveness and generally good acceptability. Outcomes were less satisfactory in patients with recurrent dislocation associated with cerebrovascular disease.

## Data Availability

No new data were created or analyzed in this study. Data sharing is not applicable to this article.

## Appendix A

**Table A1:** Reports excluded after full-text assessment or supplementary eligibility clarification when full texts were unavailable.

Title	Author, Year	Identifier	Reason for Exclusion
Single Injection Technique Prolotherapy for Hypermobility Disorders of TMJ Using 25% Dextrose: A Clinical Study	Majumdar et al., 2016 [27]	PMID: 28439165	Wrong population (recurrent TMJ dislocation and subluxation not separately reported)
Efficacy of Prolotherapy for Temporomandibular Joint Dysfunction: An Interventional Clinical Study	Assiri et al., 2025 [28]	PMID: 40364487	Wrong population (general TMD, not clearly recurrent TMJ dislocation)
Evaluation of Efficacy of 10% Dextrose Prolotherapy in Management of Temporomandibular Joint Disorders: A Prospective Study	Singh et al., 2024 [29]	PMID: 39376450	Wrong population (general TMD, not clearly recurrent TMJ dislocation)
Autologous Blood Injection for the Treatment of Recurrent Temporomandibular Joint Dislocation	Yoshioka et al., 2016 [30]	PMID: 27549675	No clinical outcomes reported (protocol only)
Bilateral platelet rich plasma injections with assisted techniques for temporomandibular joint disorders	Simsek ME, 2016 [31]	DOI: 10.18621/eurj.2016.2.1.42	Wrong population (TMJ disorders/TMJ subluxations, not clearly recurrent mandibular dislocation)
Pain relief can be painful	Bindra et al., 2015 [32]	PMID: 26600706	Wrong population (recurrent dislocation secondary to mandibular nerve block)
Treatment of recurrent luxation of the temporomandibular joint with botulinum toxin	Gilles et al., 2000 [33]	PMID: 11103426	Full text not accessible (eligibility could not be confirmed)
Treatment of habitual dislocation of the temporomandibular joint with subsynovial injection of sclerosant through arthroscope	Qiu et al., 1989 [34]	PMID: 2631116	Full text not accessible (eligibility could not be confirmed)
Comparison of mean decrease in mouth opening by autologous blood injection in superior joint space with and without pericapsular tissue in treatment of chronic recurrent temporomandibular joint dislocation in Mayo Hospital Lahore	Bukhari et al., 2020 [35]	PMID: 33341823	Full text not accessible (eligibility could not be confirmed)
Pericapsular autologous blood injection as therapy for habitual temporomandibular joint luxation	Jacobi-Hermanns et al., 1981 [36]	PMID: 6940747	Full text not accessible (eligibility could not be confirmed)
Long-term effectiveness of arthroscopic subsynovial sclerotherapy and suturing on recurrent dislocation of the temporomandibular joint	Yang et al., 1999 [37]	PMID: 11776539	Wrong intervention: combined surgical-arthroscopic treatment; injectable effect not isolable.
Effectiveness of Electromyography Guided Botulinum Toxin Injection and Dextrose Prolotherapy in Recurrent TMJ Dislocation (Radiographic and Clinical Study)	Moussa et al., 2022 [38]	DOI: 10.53730/ijhs.v6nS6.11501	Wrong intervention: injectable treatment was not exclusively intra-articular, and the effect of the intra-articular component could not be isolated.
The efficacy of dextrose prolotherapy for temporomandibular joint hypermobility: a preliminary prospective, randomized, double-blind, placebo-controlled clinical trial	Refai et al., 2011 [39]	PMID: 21757278	Wrong population: TMJ hypermobility with painful subluxation or dislocation; no stratified data for recurrent mandibular dislocation.
Injectable Agents Versus Surgery for Recurrent Temporomandibular Joint Dislocation	Renapurkar SK, Laskin DM., 2018 [40]	PMID: 29866448	Full text not accessible (eligibility could not be confirmed)
Autologous blood injection for the treatment of chronic recurrent TMJ dislocation: the prospective 6 months follow up study	Machon et al., 2015 [41]	DOI: 10.1016/j.ijom.2015.08.684	Wrong study design: conference abstract/not full original clinical study.
Short-term results of autologous blood injection for treatment of habitual TMJ luxation	Triantafillidou et al., 2012 [42]	DOI: 10.1097/SCS.0b013e31824dba9e	Wrong population: study distinguishes habitual luxation from recurrent dislocation and includes patients with habitual TMJ luxation rather than a clearly defined recurrent mandibular dislocation population.
Treatment of recurrent dislocation of the temporomandibular joint with botulinum toxin: an alternative approach	Kotimäki J, Saarinen A., 2011 [43]	PMID: 22073541	Wrong intervention: intramuscular botulinum toxin injections, not intra-articular injection
Traitement de la luxation temporo-mandibulaire récidivante par toxine botulinique	Gilles et al., 2000 [33]	PMID: 11103426	Wrong intervention: intramuscular botulinum toxin injections, not intra-articular injection
Injectable Platelet-Rich Fibrin: A Promising First-Line Therapy for Temporomandibular Joint Hypermobility	Aishwarya K, Rai M., 2024 [44]	PMID: 41648689	Wrong population: study focuses on TMJ hypermobility and includes patients with a tendency toward subluxation/dislocation; not clear whether all participants had recurrent mandibular/TMJ dislocation.
Autologous blood injection into the articular cavity for the treatment of recurrent temporomandibular joint dislocation: a case report	Kato et al., 2007 [45]	PMID: 17928731	Wrong intervention: post-procedural mandibular immobilization used.
Comparison of Autologous Blood Injection and Dextrose Prolotherapy in the Treatment of Chronic Recurrent Temporomandibular Dislocation: A Randomized Clinical Trial	Chhapane et al., 2024 [46]	PMID: 38601242	Wrong intervention: post-procedural mandibular immobilization used.
Reclamation Autohaemotherapy For Chronic Recurrent Tmj Dislocation In Mentally Challenged Young Adult: Case Report	Hange V, 2020 [47]	ORCID ID: 0000-0001-8628-3993	Wrong intervention: post-procedural mandibular immobilization used.
Autologous blood injection to the temporomandibular joint: magnetic resonance imaging findings	Candirli et al., 2012 [48]	PMID: 22474643	Wrong intervention: post-procedural mandibular immobilization used.
Evaluation of Intermaxillary Fixation (IMF) Screw Therapy with Craniomandibular Index Analysis for Chronic Recurrent Dislocation in the Temporomandibular Joint	Ertaş et al., 2022 [49]	PMID: 35046189	Wrong intervention: post-procedural mandibular immobilization used.
Treatment of recurrent TMJ dislocation in geriatric patient by autologous blood—A technique revisited	Gupta et al., 2013 [50]	PMID: 25737879	Wrong intervention: post-procedural mandibular immobilization used.
Ultrasound-guided autologous blood injection in patients with chronic recurrent temporomandibular joint dislocation	Gagnani et al., 2020 [51]	PMID: 33041574	Wrong intervention: post-procedural mandibular immobilization used.
Autologous blood injection for treatment of recurrent temporomandibular joint dislocation	Hasson O, Nahlieli O., 2001 [52]	PMID: 11598572	Wrong intervention: post-procedural mandibular immobilization used.
Arthroscopic findings after autologous blood injection in the treatment of recurrent temporomandibular joint dislocation	Matsumoto et al., 2015 [53]	DOI: 10.1016/j.ajoms.2013.12.004	Wrong intervention: post-procedural mandibular immobilization used.
Autologous blood injection as a new treatment modality for chronic recurrent temporomandibular joint dislocation	Daif ET, 2010 [54]	PMID: 19926503	Wrong intervention: post-procedural mandibular immobilization used.
Treatment of chronic recurrent dislocation of the temporomandibular joint with injection of autologous blood alone, intermaxillary fixation alone, or both together: a prospective, randomised, controlled clinical trial	Hegab AF, 2013 [55]	PMID: 23684013	Wrong intervention: post-procedural mandibular immobilization used.
Conservative treatment of recurrent temporomandibular joint dislocation with autologous blood injection	Koparal M, 2016 [56]	DOI: 10.5455/jtomc.2016.05.058	Wrong intervention: post-procedural mandibular immobilization used.
Comparative Analysis of Autologous Blood Injection and Conservative Therapy for the Management of Chronic Temporomandibular Joint Dislocation	Shah et al., 2022 [57]	DOI: 10.4103/jiaomr.jiaomr_199_21	Wrong intervention: post-procedural mandibular immobilization used.
The use of autologous blood and adjunctive ‘face lift’ bandage in the management of recurrent TMJ dislocation	Pinto et al., 2009 [58]	PMID: 19243867	Wrong intervention: post-procedural mandibular immobilization used.
Autologous blood injection for the treatment of chronic recurrent temporomandibular joint dislocation	Machon et al., 2009 [59]	PMID: 19070756	Wrong intervention: post-procedural mandibular immobilization used.
Autologous blood injection for the treatment of recurrent mandibular dislocation	Coser et al., 2015 [12]	PMID: 26022511	Wrong intervention: post-procedural mandibular immobilization used.
Comparison of autologous blood prolotherapy and 25% dextrose prolotherapy for the treatment of chronic recurrent temporomandibular joint dislocation on the basis of clinical parameters: A retrospective study	Pandey et al., 2022 [60]	PMID: 36683930	Wrong intervention: post-procedural mandibular immobilization used.
The effect of chronic temporomandibular joint dislocation: frequency on the success of autologous blood injection	Candirli et al., 2013 [61]	PMID: 24431880	Wrong intervention: post-procedural mandibular immobilization used.

**Table A2:** Characteristics of included primary studies.

Author, Year	Study Design	Population	Sample Size	Injectable Substance	Injection Site	Follow-up	Outcomes	Main Findings
Zhou et al., 2014 [17]	prospective case series	patients with non-neurogenic recurrent TMJ dislocation	45 patients	50% dextrose, preceded by 2% lignocaine	Single-site posterior periarticular injection: 2 mL 2% lignocaine followed 10 min later by 2 mL 50% dextrose. Injection deposited around the condylar neck/posterior periarticular tissues	scheduled follow-ups at 4 weeks, 3 months, and 6 months	TMJ hypermobility, maximal mouth opening, recurrence of dislocation/subluxation, clicking, adverse effects, and need for repeated injections	The authors reported improved dislocation episodes and clicking after injection, with 41/45 patients having no further dislocation or subluxation for >6 months. Most successful cases required one injection; no serious complications were reported
Machon et al., 2018 [18]	double-blind prospective study	Patients with unilateral chronic recurrent temporomandibular joint dislocation	40 patients	Autologous blood	Group A: 2 mL into the superior articular cavity + 1 mL extra-articularly during needle removal. Group B: 1 mL extra-articularly into the capsule only; no intra-articular blood injection.	follow-ups at 1, 3, 6, and 12 months	Presence of dislocations, maximum interincisal opening, pain on VAS, sound phenomena, and follow-up X-ray for range of motion and bone changes	Intra- and peri-articular ABI is more effective than peri-articular blood application alone in the treatment of CRTMD, although the difference was not statistically significant.
Xu et al., 1992 [19]	clinical case series	patients with recurrent TMJ dislocation	105 patients	5% sodium morrhuate/sclerosing agent	Extra-articular injection of sclerosing agent around the TMJ capsule/condylar neck region; not injected into the joint cavity. Dose appears to be small-volume injections, around 0.5–1 mL per injection point. Treatment could be repeated if dislocation persisted.	At least 2 years for all patients; some cases had much longer follow-up	Recurrence of dislocation, treatment effectiveness graded as excellent/good/poor, mouth opening, and complications/adverse effects.	Authors reported high clinical effectiveness: excellent in 94 cases, good in 3, poor in 8, with an excellent/good rate of 92.5%. They state that the method was simple and generally well accepted, but note lower satisfaction in patients with recurrent dislocation associated with cerebrovascular disease.
Oshiro et al., 2014 [20]	Prospective clinical study	Patients diagnosed with recurrent TMJ dislocation	14 patients	Autologous blood	Approximately 3 mL of blood was injected into the superior compartment and approximately 2 mL into the pericapsular tissue (anterior, posterior, and outside).	1 year clinical follow-up for recurrence; MRI at 1 h, 4 weeks, and 12 weeks after ABI	Recurrence of TMJ dislocation; MRI findings after ABI, including hematoma/joint effusion-like findings, T2 signal around the TMJ capsule, and decreased range of condylar movement	No recurrent TMJ dislocations were reported after 1 year. MRI suggested restricted anterior condylar movement after ABI, and authors concluded that ABI around the TMJ capsule appears to represent a very effective and safe treatment.
Yoshida et al., 2018 [21]	Retrospective clinical study	Patients with recurrent dislocation of the TMJ	21 patients	Autologous blood	Temporomandibular joint region; the discussion specifies injection into the superior compartment of the TMJ and pericapsular/periarticular tissue	Mean follow-up from first injection: 64 months; main outcome assessed within 36 months after first injection	Recurrence within 36 months after first injection, later recurrence, and final clinical outcome	Most patients remained recurrence-free: 18/21 had no recurrence within 36 months after first ABI. Authors concluded that ABI was safe and effective for recurrent TMJ dislocation, especially in patients unsuitable for mouthpiece therapy or surgery.

**Table A3:** Mapping and reference-checking sources.

Author, Year	Publication Type	Scope/Objective	Injectable Interventions Discussed	Population/Condition	Relevance to This Mapping Review	Use in This Review
Abrahamsson et al., 2020 [62]	Systematic literature review	Evaluation of surgical and nonsurgical treatment methods for temporomandibular joint luxation/dislocation.	Autologous blood injection; dextrose prolotherapy.	Temporomandibular joint luxation/dislocation, including recurrent TMJ luxation/dislocation.	Directly relevant, as it evaluates treatment methods for TMJ luxation/dislocation and includes minimally invasive injectable approaches.	Background, contextualization, comparison with previous evidence synthesis, and reference checking.
Cecílio SC, 2019 [63]	Master’s dissertation/systematic review	Identification of treatment methods with the strongest scientific evidence for recurrent temporomandibular joint luxation/dislocation.	Autologous blood injection; dextrose 50% with lidocaine.	Recurrent temporomandibular joint luxation/dislocation.	Directly relevant, as it reviews treatment methods for recurrent TMJ luxation/dislocation and includes injectable/minimally invasive approaches.	Background, contextualization, comparison with previous evidence synthesis, and reference checking.
Renapurkar SK, Laskin DM, 2018 [40]	Narrative review/clinical review	Critical evaluation of the current literature on injectable agents and surgical techniques for recurrent temporomandibular joint dislocation, with evidence-based treatment recommendations.	Sclerosing agents; dextrose prolotherapy; autologous blood injection; botulinum toxin injection.	Recurrent temporomandibular joint dislocation, including related terminology such as luxation, subluxation, open lock, and hypermobility.	Directly relevant, as it specifically compares injectable agents with surgical approaches for recurrent TMJ dislocation and discusses several injectable treatment options.	Background, contextualization, terminology clarification, and reference checking.
Nagori et al., 2018 [26]	Systematic review and meta-analysis	Assessment of the efficacy of dextrose prolotherapy compared with placebo for temporomandibular joint hypermobility.	Dextrose prolotherapy.	Patients with painful temporomandibular joint hypermobility, including subluxation or dislocation.	Directly relevant, as it evaluates dextrose prolotherapy as an injectable treatment for TMJ hypermobility and includes outcomes related to subluxation/dislocation frequency.	Background, contextualization, comparison with previous evidence synthesis, and reference checking.
Chęciński et al., 2023 [11]	Systematic review with quantitative synthesis	Identification and assessment of clinical studies evaluating autologous blood injection for the treatment of temporomandibular joint hypermobility.	Autologous blood injection.	Mandibular/TMJ hypermobility with dislocation episodes, including recurrent TMJ dislocation/luxation.	Directly relevant, as it evaluates autologous blood injection for TMJ hypermobility and includes outcomes related to recurrent dislocation episodes.	Background, contextualization, comparison with previous evidence synthesis, and reference checking.
Turosz et al., 2024 [22]	Overview of systematic reviews	Collection and comparison of systematic reviews on temporomandibular joint injection treatment and lavage.	Hyaluronic acid; autologous blood/unprocessed blood; blood-derived products; corticosteroids; hypertonic dextrose; local anesthetics; platelet-rich plasma/platelet-rich fibrin; lavage/arthrocentesis-related injectable protocols.	Temporomandibular disorders treated with TMJ injections or lavage, with a dedicated analysis of recurrent TMJ dislocation/TMJ hypermobility.	Relevant as a broad overview of systematic reviews on TMJ injections and lavage, with a dedicated section and comparison matrix for recurrent TMJ dislocation/TMJ hypermobility.	Background, contextualization, comparison with previous evidence synthesis, and reference checking.
Zhou et al., 2025 [23]	Systematic review and meta-analysis	Evaluation of the efficacy of dextrose prolotherapy for temporomandibular joint hypermobility.	Dextrose prolotherapy.	Patients with temporomandibular joint hypermobility, including recurrent TMJ dislocation/luxation.	Directly relevant, as it synthesizes evidence on dextrose prolotherapy, an injectable intervention used for TMJ hypermobility and recurrent dislocation/luxation episodes.	Background, contextualization, comparison with previous evidence synthesis, and reference checking.
Hoppe et al., 2025 [4]	Systematic review	Identification of therapeutic approaches for recurrent temporomandibular joint dislocation in pediatric patients and evaluation of their effectiveness.	Botulinum toxin injection; autologous blood injection.	Pediatric patients younger than 18 years with recurrent temporomandibular joint dislocation.	Relevant as a pediatric-focused systematic review on recurrent TMJ dislocation that includes minimally invasive injectable approaches, particularly botulinum toxin and autologous blood injection.	Background, contextualization, pediatric-specific evidence mapping, and reference checking.

**Table A4:** JBI Critical Appraisal Checklist for Case Series.

JBI Critical Appraisal Checklist for Case Series	Zhou et al. 2014 [17]	Xu et al. 1992 [19]	Oshiro et al. 2014 [20]	Yoshida et al., 2018 [21]
Were there clear criteria for inclusion in the case series?	Yes	Unclear	Yes	Unclear
Was the condition measured in a standard, reliable way for all participants?	Yes	Yes	Yes	Yes
Were valid methods used for identification of the condition for all participants included in the case series?	Yes	Yes	Yes	Yes
Did the case series have consecutive inclusion of participants?	Yes	Unclear	Yes	Unclear
Did the case series have complete inclusion of participants?	Yes	No	Yes	Unclear
Was there clear reporting of the demographics of the participants?	Yes	Yes	Yes	Unclear
Was there clear reporting of clinical information of the participants?	Yes	Yes	Yes	Yes
Were the outcomes or follow-up results of cases clearly reported?	Yes	Yes	Yes	Yes
Was there clear reporting of the presenting site(s)/clinic(s) demographic information?	Yes	No	Yes	Yes
Was statistical analysis appropriate?	Yes	Yes	Yes	Yes

**Table A5:** JBI Checklist for Quasi-Experimental Study [64].

JBI Critical Appraisal Checklist for Quasi-Experimental Studies	Machon et al., 2018 [18]
Is it clear what is the ‘cause’ and what is the ‘effect’?	Yes
Were the participants included in any comparisons similar?	Yes
Were the participants receiving similar treatment/care, other than the exposure?	Yes
Was there a control group?	Yes
Were there multiple measurements of the outcome both pre and post?	Yes
Was follow-up complete and if not, were differences described?	Yes
Were the outcomes measured in the same way?	Yes
Were outcomes measured in a reliable way?	Unclear
Was appropriate statistical analysis used?	Yes
